# Augmented Reality Physical Ergonomics (ARPE) Training for Manual Material Handling Workers: A Protocol for a Quasi-experimental Study

**DOI:** 10.7759/cureus.92302

**Published:** 2025-09-14

**Authors:** Fatimah Zahra Norman, Siti Munira Yasin, Mariam Mohamad, Meram Azzani

**Affiliations:** 1 Department of Public Health Medicine, Faculty of Medicine, Universiti Teknologi MARA (UiTM), Sungai Buloh, MYS

**Keywords:** augmented reality, ergonomics, manual material handling, occupational health, work-related musculoskeletal disorders

## Abstract

Introduction: Manual material handling (MMH) workers are at an increased risk of work-related musculoskeletal disorders (WMSDs) due to the physically demanding nature of their tasks. Traditional ergonomic training methods often fail to engage learners effectively. This protocol describes an Augmented Reality Physical Ergonomics (ARPE) training module that aims to enhance ergonomic knowledge, supports worker self-assessment, and encourages preventive practices through an immersive, interactive learning experience.

Methods: The ARPE module was developed with a literature review, expert input, and pilot usability testing and is delivered via a mobile XR app featuring 3D task simulations and ergonomic demonstrations. We will conduct a two-arm, six-week quasi-experimental study in manufacturing settings (n=78 MMH workers; 1:1 ARPE vs. standard Department of Occupational Safety and Health (DOSH) training). Outcomes will be measured at baseline, immediately post-training, and at six weeks. The primary outcome is ergonomic knowledge; secondary outcomes are self-prevention behaviors and self-evaluation. Group differences across time will be evaluated using repeated-measures methods. A separate usability assessment of the ARPE app employed a validated mHealth usability questionnaire in an independent worker sample.

Results: As this is a study protocol, results are not yet available. The study is designed to assess changes in ergonomic knowledge (primary outcome) and self-prevention behaviors and self-evaluation (secondary outcomes). Data collection and analysis are planned through November 2025.

Ethics and trial registration: The study was approved by the Research Ethics Committee of Universiti Teknologi MARA (UiTM) (approval number: REC/11/2024 (PG/MR/550)) and is registered with ClinicalTrials.gov (US National Library of Medicine) under the identifier NCT06993298.

Conclusion: This protocol describes an augmented reality (AR)-based ergonomic training module (ARPE) that aims to provide an innovative, expert-validated, immersive approach to training. The study will evaluate effects on ergonomic knowledge (primary), self-prevention behaviors, and self-evaluation among MMH workers. Findings may support the use of AR as a scalable occupational health training tool and may inform strategies to reduce work-related musculoskeletal risk.

## Introduction

Work-related musculoskeletal disorders (WMSDs) are a major cause of occupational health problems and lost productivity worldwide, especially among manual material handling (MMH) workers who regularly lift, lower, push, pull, or carry loads [1-3]. In Malaysia, WMSDs pose significant health and economic challenges, as shown by risk patterns among industrial workers and records of compensation claims [4,5]. Ergonomics training is essential for prevention; however, traditional methods, such as lectures, static videos, and one-way briefings, often lack interactivity, practical application, and immediate feedback. These limitations lead to poorer knowledge retention, limited skill development, and less effective behavior change [6-8].

Augmented reality (AR) in ergonomic learning involves overlaying digital content onto real tasks, enabling workers to visualize risk factors (e.g., awkward postures, load paths), receive instant cues, and practice safer techniques on-site. By combining task-specific guidance with real-time feedback, AR aims to improve understanding, support self-assessment, and promote preventive actions during MMH activities [9-11]. In safety-critical fields such as construction, manufacturing, healthcare, and aviation, AR/virtual reality (VR) training has been shown to increase engagement, procedural accuracy, postural awareness, and knowledge transfer compared to traditional passive methods [7,10,12]. However, comprehensive evaluations focusing on MMH ergonomics in Malaysia aligned with national guidelines and measuring ergonomic knowledge (primary), along with self-preventive behaviors and self-assessment (secondary), using quasi-experimental designs are still limited [4,11,13,14]. On the other hand, studies highlight the positive impact of AR on cognitive skill acquisition, learning transfer, and real-time error correction among workers in physically demanding roles [15]. Workers also report higher engagement levels and motivation to participate in repeated training sessions when AR is used [16]. Thus, this study aims to develop and evaluate an Augmented Reality Physical Ergonomics (ARPE) training module for MMH workers by aligning immersive, task-level content with local ergonomic guidelines, specifically, to design, develop, and content validate the ARPE module; determine its usability among MMH workers; and test its effectiveness in a two-arm intervention (ARPE versus guideline-based control). The primary effectiveness objective is to determine whether ARPE yields a greater increase in ergonomic knowledge from baseline (t_0_) to immediate post-training (t_1_) than guideline-based training, while secondary objectives are to assess whether ARPE improves self-prevention behaviors and self-evaluation at t1 and at six-week follow-up (t_2_). The primary goal is to determine if ARPE leads to a greater increase in ergonomic knowledge from baseline to immediate post-training (t_0_→t_1_) compared to guideline-based training. The secondary goals are to assess whether ARPE improves self-prevention behaviors and self-evaluation at t_1 _and during the six-week follow-up (t_2_). If proven effective, ARPE could complement traditional training and promote safer work practices, although longer-term outcomes (such as WMSD incidence and productivity) are beyond the scope of this protocol.

## Materials and methods

This study used a mixed-methods approach combining both qualitative and quantitative techniques across two main phases. The aim was to develop, validate, and evaluate an ARPE training module for MMH workers. In Phase 1, the focus was on designing and validating the AR content, platform, and assessment tools, followed by usability testing. Phase 2 involved a quasi-experimental intervention to measure the module's impact on ergonomic knowledge, self-prevention behaviors, and self-evaluation skills.

Approval of ethics

This study received approval from the Research Ethics Committee of Universiti Teknologi MARA (UiTM) (approval number: REC/11/2024 (PG/MR/550)). It will be conducted in accordance with the Declaration of Helsinki and the Malaysian Good Clinical Practice Guideline. All participants will receive a study information sheet and provide written informed consent before any procedures. Participant privacy will be protected via deidentification (coded IDs), with direct identifiers stored separately from research data. Electronic files will be maintained on secure, password-protected, access-restricted institutional servers; any hard-copy materials will be kept in locked storage. Only authorized study personnel will have access to identifiable data, and results will be reported in aggregate to minimize re-identification risk. Any substantial protocol amendments will be submitted to the Research Ethics Committee and communicated to relevant stakeholders.

Phase 1a: development and content validation of the ARPE training module

In Phase 1a, the fuzzy Delphi method (FDM) was employed to achieve expert consensus on the content of the AR ergonomic training module. Ten experts were purposively selected from diverse fields, including public health, occupational health, ergonomics, and instructional design in educational technology. This phase involved online interviews to validate training content based on relevant literature and established ergonomic guidelines. Structured rounds of questionnaires using a 7-point Likert scale were administered to refine the content, ensuring methodological rigor and reducing subjectivity [17]. Expert consensus was established by achieving at least 75% agreement and a d-value (d-construct) threshold of ≤0.2, per the accepted FDM criteria [18]. A defuzzification process was then conducted to rank the importance of each item based on expert scoring. These procedures enhanced the scientific credibility and contextual relevance of the final training content, which was structured around three primary learning domains: ergonomic knowledge, self-prevention, and self-evaluation. The proposed items were compiled into a module guided by these expert-reviewed constructs.

ARPE Training Module Platform Selection

The AR was developed in the MAKAR XR platform, chosen for its user-friendly interface, cross-platform support, cost-effectiveness, and proven utility in educational and training settings [19]. MAKAR XR enables the creation of AR, VR, and mixed reality (MR) content on iOS, Android, and web, allowing educators and developers to design immersive simulations without complex coding. The ARPE module featured scenario-based simulations with 3D models showing proper MMH, body posture, and ergonomic tool use. Users interact with digital objects, receive audiovisual feedback, and refine ergonomic practices in realistic settings. The module was optimized for mobile, with an intuitive interface to boost engagement and learning. The instructional designer was a certified professional technologist with expertise in instructional design, ergonomics, and AR training systems. The platform was chosen independently based on research needs, with no influence from financial or institutional interests.

Assessment Tool Development and Validation

To evaluate the ARPE module, we developed a multi-domain assessment battery that covers ergonomic knowledge (primary) and self-prevention and self-evaluation (secondary). The knowledge test was aligned with occupational ergonomics guidelines and translated into Malay (scored 0-100%). The behavioral instruments were adapted from Diego-Mas et al. and operationalized through PQ (Perceived Risk; 11 items: Concern, Control, Loads, Repetitiveness, Postures), RQ (Reaction; 6 items: Prior AR Exposure, Expectation, Interest, Usefulness), and SA (Self-Assessment; 7 items: Memory, Concern, Control), all using 7-point Likert scales with domain scores calculated as item means (higher scores indicate a greater presence of the construct) [14]. Translation and cultural adaptation were followed by assessments of content validity (Content Validity Index (CVI); 7 experts), face validity (Face Validity Index (FVI); 10 MMH workers), construct validity (Exploratory Factor Analysis (EFA); n=100), and reliability testing. For CVI, we applied standard acceptability criteria (Item-level Content Validity Index (I-CVI) ≥0.78; Scale-level Content Validity Index, average method (S-CVI/Ave) ≥0.90), consistent with recommended procedures in content validity studies [20]. For factorability, we required Kaiser-Meyer-Olkin (KMO) >0.60 and Bartlett's test p<0.05, with primary loadings ≥0.40. Internal consistency was considered acceptable at Cronbach's α ≥0.70, good at ≥0.80, and excellent at ≥0.90 (with corrected item-total correlations ≥0.30). Test-retest reliability was assessed using the intraclass correlation coefficient (ICC) (two-way, absolute agreement), with the following thresholds: <0.50 poor, 0.50-0.74 moderate, 0.75-0.89 good, and ≥0.90 excellent. 95% confidence intervals (CIs), standard error of measurement (SEM), and minimal detectable change at the 95% confidence level (MDC_95) will also be reported. In prior related studies, the scales demonstrated α coefficients ranging from 0.71 to 0.92 [14]. All analyses will be performed using IBM SPSS Statistics for Windows, Version 29.0 (IBM Corp., Armonk, New York, United States).

Phase 1b: usability outcome (ARPE (AR) mobile app usability)

Usability will be evaluated using the Malay version of the Mobile Health App Usability Questionnaire (M-MAUQ) [21]; its conceptual framework and item pool are grounded in the original English MAUQ development and validation by Zhou et al. [22]. The 18-item instrument includes three domains, namely, ease of use (5 items), interface satisfaction (7 items), and usefulness (6 items), rated on a 7-point Likert scale (1 (strongly disagree) to 7 (strongly agree)). Acceptable usability is defined beforehand as a mean item score above 4.0 (i.e., above the neutral midpoint), which corresponds to a total score of 72 or higher out of 126 (18×4). This threshold indicates a clear tendency toward positive usability on a 7-point scale and is commonly used for MAUQ-based app evaluations when no specific clinical cut-points are available. For better understanding, we will also report categories at the domain level: minimal acceptable (>4.0 to <5.0), good (≥5.0 to <6.0), and excellent (≥6.0). Phase 1b will use a cross-sectional, post-use design with an independent sample of approximately 100 MMH workers. Participants will receive a brief orientation and a guided first use of the ARPE app and then complete the M-MAUQ immediately afterward in the same session. Regarding feedback, we will calculate mean scores for each domain and item; any item with a mean score of 4.0 or less, a corrected item-total correlation below 0.30, or recurring free-text concerns will be flagged for revision. Results will be compiled into an action log (UI issues, content clarity, navigation), prioritized by severity and frequency, and addressed quickly before starting the Phase 2 quasi-experimental study. Reliability (Cronbach's α; optional test-retest ICC in a subsample) will be reported according to the thresholds specified in the Assessment Tool Development and Validation subsection.

Phase 2: evaluation of the AR ergonomic training module's effectiveness

This Phase 2 evaluation employs a two-arm, parallel-group, quasi-experimental design among warehouse MMH workers. To minimize cross-group contamination at the worksite, allocation is non-randomized at the site level to (i) ARPE training (intervention) or (ii) standard guideline-based training (control) in approximately a 1:1 ratio. We will report baseline comparability between groups in terms of demographics, job tenure, department/shift, prior ergonomic training, and baseline outcomes. Any imbalances will be addressed analytically.

Eligibility Criteria

This study will involve Malaysian citizens employed as MMH workers in warehouses. Participants shall be at least 18 years old and regularly engage in MMH tasks such as lifting, lowering, pushing, pulling, carrying, holding, or restraining objects that exceed 14 kilograms, according to the National Institute for Occupational Safety and Health (NIOSH) guidelines 2015. They must work at least eight hours daily and perform these tasks as part of their routine responsibilities. Additionally, participants must have a mobile phone capable of downloading and using the MAKAR XR app. Those excluded from participation include pregnant individuals, anyone who has attended ergonomic training within the past six months, and interns or temporary workers.

Setting and Recruitment

The study takes place at two warehouses in the Klang Valley. We recognize differences between sites, such as task variety, shift schedules, and prior ergonomic training, which could influence the results. Recruitment is coordinated with each company's safety and health committee through invitation emails and letters, workplace posters, e-posters in departmental WhatsApp groups, and targeted phone calls. Interested workers are screened based on specific inclusion and exclusion criteria and are asked to provide their consent individually.

Allocation

To reduce cross-group contamination, allocation is non-randomized at the site/shift level: one warehouse is assigned to ARPE (intervention) and the other to guideline-based training (control). The site closer to HQ was pragmatically chosen for the intervention to ease monitoring; the second site serves as the control. The sites are geographically separated to minimize contact between groups. We acknowledge that differences between sites may confound the results; therefore, we will (i) document site characteristics (department mix, average load profiles, previous musculoskeletal disorder (MSD) initiatives), (ii) report baseline comparability using standardized mean differences (SMDs), and (iii) adjust analyses as described below.

Sample Size

The sample size was determined from a previous study, which indicated a 57.5% improvement in knowledge [23]. In this study, 52.5% of participants demonstrated very good self-efficacy, while 85% reported perfect practice following the ergonomic training program (ETP) in the experimental group. This calculation utilized a 95% CI and an 80% power. The calculated sample size required was 28 participants per group. To account for a 40% attrition rate [24], an additional 11 participants were included, resulting in a final sample size of 39 participants per group informed by reports of high turnover in shift-based workforces [24]. Therefore, the total number of participants required for this study is 78, ensuring sufficient power to detect significant differences between the intervention and control groups while accounting for potential participant dropout.

Intervention

**Figure 1:**
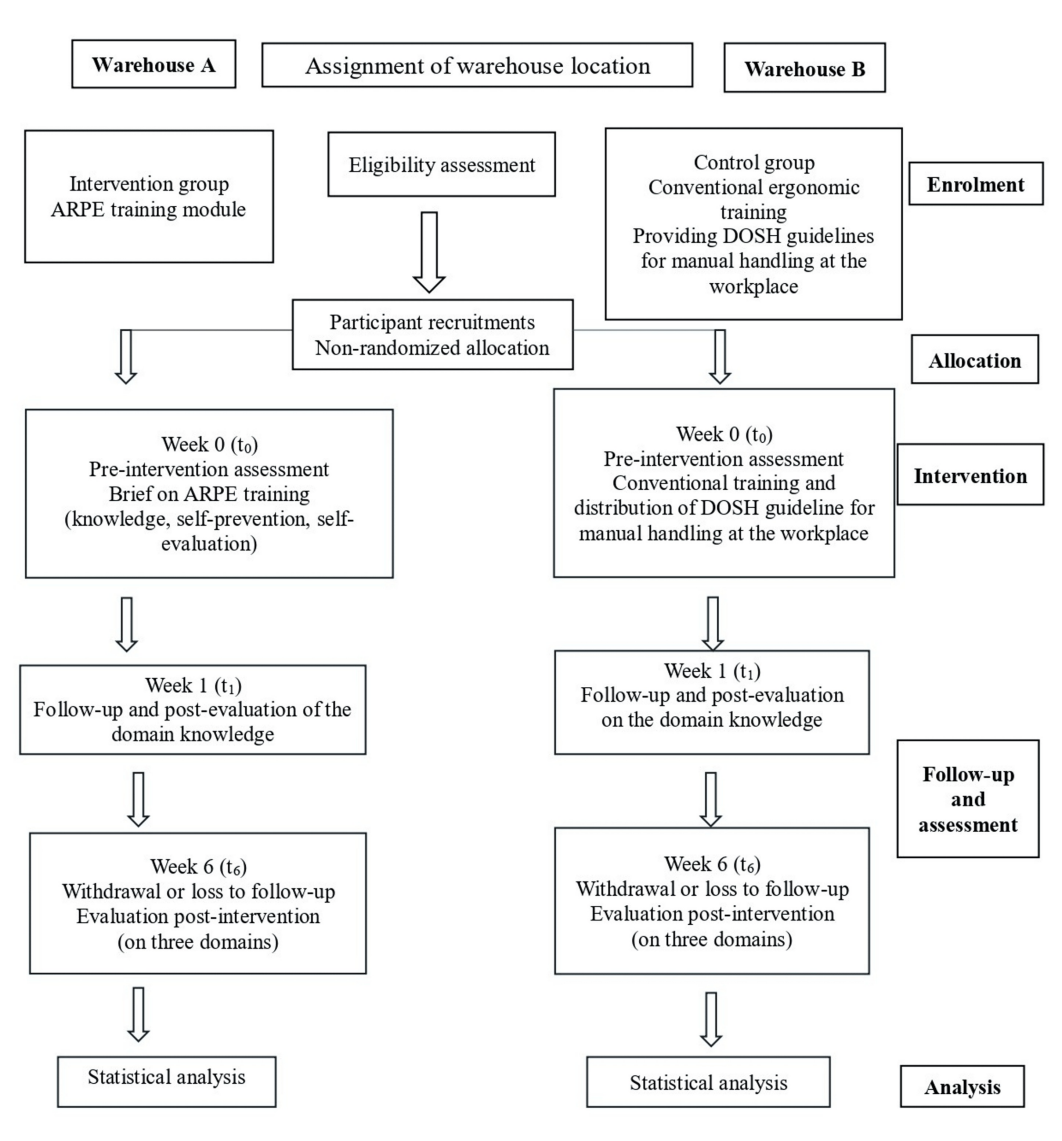
Intervention study process flow ARPE: Augmented Reality Physical Ergonomics; DOSH: Department of Occupational Safety and Health

The study aims to enhance workers' understanding of ergonomics, increase awareness of WMSDs, promote a culture of workplace safety and health, and encourage the adoption of effective ergonomic practices. The ARPE module is based on scientific evidence, including literature, manuals, and guidelines aligned with the Department of Occupational Safety and Health (DOSH) for MMH workers. Its structure, which includes three domains and 25 subdomains, is summarized in Table 1. Operational steps are shown in Figure 1: eligibility screening and recruitment by warehouse; non-randomized, site-level assignment (Warehouse A: ARPE; Warehouse B: conventional training); baseline assessment at week 0; immediate post-knowledge assessment at week 1; and a follow-up evaluation at week 6 across all three domains, with withdrawals/losses to follow-up recorded. Participants in the intervention and control groups were recruited from separate warehouses to minimize cross-contamination, and subsequent statistical analyses were conducted as outlined in Figure 1.

**Table 1:** Domains, variables, instruments/tools, timepoints, and purposes of assessment in the ARPE study t_0_: baseline (pre-intervention); t_1_: week 1 (post-knowledge evaluation); t_2_: week 6 (final follow-up) PQ: Perceived Risk; RQ: Reaction; SA: Self-Assessment; ARPE: Augmented Reality Physical Ergonomics

Domain	Variable	Instrument/tool	Timepoints	Purpose
Baseline characteristics	Age, gender, ethnicity, education level, job category, years of service, shift work, work process	Self-administered questionnaire (Malay version)	t_0 _ (week 0: before intervention)	To describe participant demographics and work-related characteristics
Primary outcome	Ergonomic knowledge	Adapted Diego-Mas et al.'s PQ Questionnaire [14]	t_0_, t_1_,t_2_	To evaluate knowledge acquisition and the retention of ergonomic principles
Secondary outcome 1	Self-prevention	Adapted Diego-Mas et al.'s PQ Questionnaire [14]	t_0_, t_1_, t_2_	To assess the implementation of preventive ergonomic behaviors
Secondary outcome 2	Self-evaluation	Adapted Diego-Mas et al.'s RQ and SA Questionnaires [14]	t_0_, t_1_, t_2_	To assess awareness and self-assessment of ergonomic risks and practices

Introduction phase (t0): The intervention group will receive a briefing on AR physical ergonomic training and download the MAKAR XR app, which includes instructions to familiarize them with its interface and tasks. The control group will undergo traditional ergonomic training, which will begin with a briefing and lectures based on the DOSH MMH guidelines [25]. This training will focus on preventing ergonomic injuries and MSDs, emphasizing the importance of taking breaks, using adjustable tools, proper lifting techniques, maintaining good posture, and conducting posture assessments. It will cover reporting WMSD symptoms, consulting healthcare providers, and ergonomic hazard management, promoting safe task performance, and adherence to health guidelines. Self-evaluation will assess MMH techniques, WMSD risk factors, potential hazards, and control initiatives such as stretching. This session will last 30-45 minutes and will include baseline assessments on knowledge, self-prevention, and self-evaluation for both groups.

Intervention phase (t1): This phase assesses knowledge acquisition and retention through questionnaires. Participants receive WhatsApp reminders to engage with AR content in the MAKAR XR apps and adhere to the MMH guidelines. They should view the ARPE module training within two to three days and can provide feedback to the researcher via WhatsApp. Regular WhatsApp check-ins and automated reminders will track participant engagement and potential dropouts. Weekly or bi-weekly check-ins will be conducted to inquire about participants' progress and address any issues. Missing multiple reminders will prompt additional follow-up to address the potential problems with dropouts.

Post-intervention phase (t2): In the sixth week, a follow-up and post-evaluation of the training will occur. Post-evaluation on the domains of self-prevention and self-evaluation will be assessed by disseminating questionnaires to the participants. This phase aims to measure the long-term retention of ergonomic knowledge and the practical application of self-prevention and self-evaluation techniques learned during the training. Data collected will be analyzed to determine the effectiveness of the AR training module compared to the conventional training method.

Quality control measures will be implemented to minimize study contamination, with participants instructed not to share the messages they received. Recruitment efforts ensure that participants from both the intervention and control groups are drawn from distinct warehouse locations. Recruitment focuses on workers who willingly agree to commit to the study, aiming to enhance compliance with the intervention. Participants are advised to adhere strictly to the intervention procedure, which involves actively observing and comprehending each aspect of the ARPE training intervention throughout the designated study period. To ensure compliance with the protocol, participants will be instructed not to share details of the intervention and must follow the ARPE training guidelines. Adherence will be monitored through regular WhatsApp check-ins and confirmation from supervisors. Supervisors will be asked to verify their employees' participation, emphasizing the importance of the intervention and fostering a supportive work environment. Instances of non-compliance, such as missed sessions, will lead the researcher to reach out, offering assistance and encouraging continued participation, thereby supporting the successful implementation of the intervention. This strategy is anticipated to greatly enhance the overall effectiveness of the study by keeping participants engaged and compliant with the training protocols. Participants received weekly reminders, and adherence was monitored through supervisor sign-offs and app analytics. Outcome assessors and analysts remained blinded to allocation.

## Results

Planned outcomes and data analysis

Since this is a protocol, no results are available yet. The primary outcome is ergonomic knowledge, while secondary outcomes include self-prevention behaviors and self-evaluation, measured at baseline (T0), post-training (T1), and six weeks later (T2). Analyses will follow intention-to-treat principles using repeated-measures mixed models (fixed effects: group, time, group×time), with site effects and adjustments for baseline scores and prespecified covariates (e.g., age, tenure, department/shift, prior training). Two-sided α=0.05; results will be reported as adjusted mean differences with 95% CI. Sensitivity analyses will incorporate site-clustered standard errors (SEs), a random intercept for the site if applicable, and inverse-probability-of-treatment weighting. Outcome assessors and the statistician will remain blinded to group allocation. 

Protocol Status and Timeline

Recruitment and baseline assessments are ongoing; follow-up continues through November 2025, after which data will be cleaned and analyzed. No interim results are available.

Interpretation of Change

A structured outcome framework was created to assess the effectiveness of the ARPE training module. The assessments are divided into three domains: ergonomic knowledge (the primary outcome), self-prevention behaviors (secondary outcome 1), and self-evaluation skills (secondary outcome 2). Each domain is evaluated at three key timepoints: baseline (t_0_, before the intervention), week 1 (t1, after the intervention to assess knowledge), and week 6 (t2, follow-up post-intervention). The tools used are adapted from Diego-Mas et al. [14]. All scales are scored so that higher scores reflect better outcomes. The primary endpoint is the change in ergonomic knowledge from baseline to immediate post-training (T0→T1). ARPE will be considered superior if the adjusted group-time effect (ARPE-control) is positive with a 95% CI that does not include 0 (two-sided α=0.05); persistence will be evaluated at T2. For secondary domains (self-prevention, self-evaluation), adjusted group×time effects at T1 and T2 will be regarded as improvements when the 95% CI excludes 0; these analyses are exploratory and supportive.

Baseline Characteristics

Baseline characteristics are summarized in Table 1: continuous variables are presented as mean (SD) or median (IQR), while categorical variables are shown as n. These include age, sex, ethnicity, education, job category, employment duration, shift work, MMH process details, and prior ergonomic training. To evaluate comparability between groups at t_0_, SMDs will be used instead of significance tests. Thresholds for SMD are as follows: <0.10 (negligible), 0.10-0.20 (small), and >0.20 (substantial imbalance). Variables with SMD >0.20, along with prespecified covariates such as age, tenure, department/shift, and prior training, will be adjusted for in the repeated-measures mixed models. These models will incorporate site effects and cluster-robust SEs, as detailed in the Statistical Analysis section. If baseline p-values are reported, they are purely descriptive and not used to determine equivalence.

Primary Outcome: Ergonomic Knowledge

Repeated measures (t_^0^_, t_1_, t_2_) will be analyzed using generalized estimating equations (GEE). The model includes group (ARPE vs. control), time, group×time interaction, site (fixed effect), and prespecified covariates (e.g., age, department/shift, prior training). The primary comparison is the adjusted ARPE-control difference at t_1_, obtained from the group×time term (with baseline accounted for by including t0 in the repeated-measures model); durability will be evaluated at t_2_. Results will be reported as marginal adjusted mean differences (95% CI; two-sided α=0.05) and SMDs. Within-group changes across (t_0_, t_1_, t_2_) will be derived from model-based marginal contrasts with 95% CI. Missing data will be managed under missing completely at random (MCAR)/missing at random (MAR) assumptions using GEE with robust SEs, with sensitivity analyses employing inverse-probability-weighted GEE and multiple imputation.

Secondary Outcome 1: Self-Prevention 

Self-prevention (PQ-derived domain mean; 7-point Likert scale; higher scores indicate better performance; scores are rescaled from 0 to 100) will be analyzed using GEE (identity link, Gaussian), clustering on participants with robust (sandwich) SEs and an exchangeable working correlation. The model includes group (ARPE vs. control), time (t_0_, t_1_, t_2_), group×time, site (fixed effect), and prespecified covariates (age, tenure, department/shift, prior training). Between-group change is tested via group×time with prespecified contrasts reporting marginal ARPE-control differences at t_1_ and t_2_ with 95% CI (two-sided α=0.05) and SMDs. Within-group change (t_1_-t_0_, t_2_-t_0_) will be presented using model-based marginal contrasts (descriptive). Missing data will be addressed under MCAR/MAR using GEE with robust SEs; sensitivity analyses will include inverse-probability-weighted GEE.

Secondary Outcome 2: Self-Evaluation 

Self-evaluation will be assessed using adapted RQ and SA domain means (7-point Likert scale; higher scores indicate better performance; scores are rescaled from 0 to 100). Repeated measures (t_0_, t_1_, t_2_) will be analyzed with GEE using an identity link and Gaussian family, clustering on participants with robust (sandwich) SEs and an exchangeable working correlation (Quasi-likelihood under the Independence model Criterion (for comparing GEE correlation structures) (QIC) to compare AR(1) and unstructured). The model includes group (ARPE vs. control), time, the interaction between group and time, site (fixed effect), and prespecified covariates (age, tenure, department/shift, and prior training). Between-group differences will be tested via the group×time interaction, with prespecified contrasts reporting marginal ARPE-control differences at t_1_ and t_2_ (95% CI; two-sided α=0.05; SMDs shown). Within-group changes (t_1_-t_0_, t_2_-t_0_) will be derived from model-based marginal contrasts (95% CI) and presented descriptively; inference will follow the GEE model.

Data Collection

Baseline data collection will occur during the participants' first training session, before their enrolment in the study. Both primary and secondary outcomes will be evaluated at three intervals: the baseline measurement, the end of the first week, and the conclusion of the sixth week. Participants will receive prompts to complete data collection at each point through direct reminders and easy survey submission options. During baseline collection, reminders will be provided at the initial training session, with notifications sent via WhatsApp or phone calls, as preferred by the participants. During recruitment, participants will fill out self-administered questionnaires using a Google Form. For follow-up assessments in the first and sixth weeks, the same communication channels will be used for reminders, and the Google Form will be the sole method for these evaluations. These reminders will be strategically timed to foster consistent engagement and compliance, thus facilitating accurate data collection throughout the study. This strategy ensures a thorough, flexible, and efficient monitoring of data collection over the two months.

Data Management

In the data management domain, we are committed to upholding the privacy and confidentiality of participants. To achieve this, each participant will be assigned a unique anonymous research identification number (ID). Participant names and data will be securely stored on a password-protected researcher's laptop, linked exclusively to their respective research ID for this study. Regular data backups will be executed and stored in a secure location. Upon the study's conclusion, data on the computer will be transferred to an external hard drive, and the computer data will be permanently erased. Both external hard drives and hardcopy data will be safeguarded in a locked office designated for researchers. The data will be retained for at least three years post-study completion, after which it will be systematically erased. This stringent data management protocol ensures the highest data security and participant confidentiality throughout and beyond the study period.

Data Analysis

Analyses will be conducted using IBM SPSS Statistics for Windows, Version 29.0 (IBM Corp., Armonk, New York, United States). After cleaning and verifying the data, descriptive statistics will summarize continuous variables as mean (SD) or median (IQR) and categorical variables as n (%). Baseline comparability will be assessed with SMDs (<0.10 negligible; 0.10-0.20 small; >0.20 meaningful); any baseline p-values are for descriptive purposes only.

Effectiveness analyses will use GEE with an identity link and Gaussian family, clustering on participants with robust (sandwich) SEs and an exchangeable working correlation; QIC will compare the exchangeable structure with AR(1)/unstructured structures. Models will include group (ARPE vs. control), timepoints (t_0_, t_1_, t_2_), group×time interactions, site (fixed effect), and prespecified covariates (age, job tenure, department/shift, prior ergonomic training). The primary comparison is the adjusted ARPE-control difference at t_1 _for ergonomic knowledge (0-100%); durability will be assessed at t_2_. Within-group changes (t_1_-t_0_, t_2_-t_0_) will be derived from model-based marginal contrasts and presented descriptively. Results will be shown as marginal adjusted mean differences with 95% CI (two-sided α=0.05) and SMDs. All analyses will follow intention-to-treat principles. Missing repeated measures will be addressed under MCAR/MAR assumptions via GEE with robust SEs; inverse-probability-weighted GEE and multiple imputation sensitivity analyses will also be reported. For multiple secondary domains and timepoints, multiplicity will be controlled using the Benjamini-Hochberg FDR (q=0.05) method, with both raw and FDR-adjusted p-values presented.

## Discussion

This protocol evaluates whether an AR-based physical ergonomics module (ARPE) improves ergonomic knowledge (primary) and self-prevention/self-evaluation (secondary) among MMH workers compared to guideline-based training. AR addresses the limitations of passive formats by increasing engagement, interactivity, and task-specific feedback, which are linked to better learning and skill transfer in professional training [26,27]. The quasi-experimental site/shift-level design aims to minimize cross-group contamination while allowing evaluation under routine working conditions [28]. Conventional classes and videos often fall short in changing behavior during high-risk tasks [7,11]. By incorporating 3D simulations and in situ cues, ARPE is expected to support deeper encoding, immediate feedback, and context-specific practice, potentially leading to safer work techniques. This study extends AR/VR training evidence to warehouse MMH ergonomics using validated tools, a prespecified analysis plan, and blinded assessment/analysis, which improves interpretability [26,27]. Strengths include alignment with occupational guidance and measures validated locally, pragmatic site/shift assignment to minimize contamination, repeated measures at T0, T1, and T2 to evaluate immediate gains and short-term durability, and a pre-planned model of group×time effects with covariate adjustment and site effects. Limitations persist. Additionally, we will perform inverse-probability-weighted sensitivity analyses. Self-report outcomes may be influenced by social desirability bias [29]; mitigation strategies include the use of anonymous, self-administered tools, blinded assessors and analysts, and aggregate reporting. Non-randomized allocation could lead to selection bias and site-level confounding [30]. We will report baseline comparability using SMDs, adjust for age, tenure, department/shift, and prior training, and include site effects. Contamination will be minimized through separate sites/shifts, instructions not to share materials, and routine schedule reminders. Expected attrition and varying digital literacy are addressed through weekly reminders, supervisor sign-offs, app usage analytics, and an intention-to-treat analysis with proper handling of missing data (MAR/MCAR). Residual confounding remains possible and will be considered in cautious interpretation.

Implications and future directions

If ARPE enhances knowledge and promotes supportive behavior changes, AR could become a scalable supplement to traditional training, especially where scheduling, geography, or literacy barriers limit classroom delivery. Future priorities include longer follow-up periods, objective task measures (e.g., posture/load monitoring), cost-effectiveness analyses, and conducting cluster-randomized or stepped-wedge trials to strengthen causal inference [28,30].

## Conclusions

This protocol outlines an AR-based ergonomics training module (ARPE) designed to enhance ergonomic knowledge (mainly) and self-prevention/self-evaluation behaviors among MMH workers. By comparing ARPE to guideline-based training, the study will determine if interactive, immersive learning provides short-term measurable benefits. If proven effective, ARPE could act as a scalable addition to traditional training to help lower WMSD risk and promote worker well-being. The results will also inform program design and implementation, such as adherence support and supervisor involvement, guide decisions on scaling in warehouse environments, and highlight priorities for future trials (longer follow-up, objective task metrics, and cost-effectiveness).
